# Selenium‐Bearing Conducting Polymer/Graphene Quantum Dot Hybrid for Enzyme Based Electrochemical Biosensor Targeting Tyrosinase Inhibition Via Rosmarinic Acid

**DOI:** 10.1002/open.70223

**Published:** 2026-05-10

**Authors:** Raghad Alhardan, Gulsu Keles, Sevki Can Cevher, Derya Altay, Panagiota M. Kalligosfyri, Sinem Aslan Erdem, Ali Cirpan, Stefano Cinti, Sevinc Kurbanoglu

**Affiliations:** ^1^ Department of Analytical Chemistry Faculty of Pharmacy, Ankara University Ankara Türkiye; ^2^ The Graduate School of Health Sciences Ankara University Ankara Türkiye; ^3^ Department of Chemistry Middle East Technical University Ankara Türkiye; ^4^ Department of Traditional Complementary and Integrative Medicine Institute of Public Health Ankara Yıldırım Beyazıt University Ankara Türkiye; ^5^ Department of Pharmacy University of Naples Federico II Naples Italy; ^6^ Department of Pharmacognosy Faculty of Pharmacy Ankara University Ankara Türkiye; ^7^ The Center for Solar Energy Research and Application (GUNAM) Middle East Technical University Ankara Türkiye; ^8^ Department of Polymer Science and Technology Middle East Technical University Ankara Türkiye; ^9^ Department of Micro and Nanotechnology Middle East Technical University Ankara Türkiye; ^10^ Bioelectronics Task Force University of Naples Federico II Naples Italy; ^11^ Sbarro Institute for Cancer Research and Molecular Medicine Center for Biotechnology College of Science and Technology Temple University Philadelphia Pennsylvania U.S.A.; ^12^ Integrated Technologies Research Center (BÜTAM) Ankara University Ankara Türkiye

**Keywords:** catechol, conducting polymers, graphene quantum dots, rosmarinic acid, tyrosinase

## Abstract

In this present study, an enzyme‐based amperometric nanobiosensor was designed and fabricated through the immobilization of tyrosinase onto a selenium‐bearing conducting polymer (poly[BDT‐alt‐(TP;BSe)]), in combination with NH_2_‐functionalized graphene quantum dots incorporating benzoselenadiazole, thienopyrroledione, and benzodithiophene moieties. The innovative nanobiosensor was developed by crosslinking the tyrosinase enzyme with the help of glutaraldehyde in a novel selenium‐bearing conducting polymer and NH_2_ functionalized quantum dots matrices. Various factors influencing the biosensor's performance were optimized, including the amount of NH_2_‐functionalized quantum dots, poly[BDT‐alt‐(TP;BSe)], tyrosinase, and glutaraldehyde. Under optimized experimental parameters, catechol detection was achieved across 0.1–88 µM with a detection limit of 0.023 µM. Subsequently, the designed biosensor is used to follow tyrosinase inhibition via rosmarinic acid‐containing plant materials, specifically *Rosmarinus officinalis*. After optimization of the inhibition conditions, I_50_ values were determined as 21 µM for *Rosmarinus officinalis*. This represents the first literature report utilizing electrochemical methodology with a novel conducting polymer coupled with NH_2_‐functionalized graphene quantum dots for tyrosinase biosensing to evaluate rosmarinic acid inhibitory effects.

## Graphical abstract

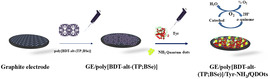

## 1 Introduction

Enzyme‐based electrochemical biosensors constitute a potent and adaptable category of analytical instruments crafted for the selective identification of various biomolecules. These biosensors utilize the catalytic efficiency of enzymes to facilitate precise biochemical reactions, thereby converting targeted analytes into measurable electrochemical signals. The fundamental approach involves immobilizing enzymes on electrode surfaces and creating a bioactive interface that responds to the presence of the target substance [1, 2, 3]. Various methods, such as physical adsorption, chemical cross‐linking, polymer matrix entrapment within polymer matrices, or covalent bonding, can be employed to immobilize enzymes onto electrodes. This immobilization maintains the enzyme's stability and functionality, enhancing the durability and overall effectiveness of the sensor. The enzymatic reaction either generates or consumes electrons, modifying the electrode's electrochemical properties including current or potential [4, 5]. These modifications are then transformed into quantifiable signals, establishing a proportional relationship between signal intensity and the concentration of the target analyte. Enzyme‐based electrochemical biosensor platforms find widespread application in clinical diagnostics, nutritional safety, and environmental assessment. They possess remarkable advantages, including superior specificity, improved sensitivity, and rapid response times [6, 7, 8, 9, 10]. Additionally, the ability to customize these sensors for various enzymes and substrates makes them versatile for a broad range of target molecules [11, 12]. The continuous advancements in enzyme immobilization techniques, integration with nanomaterials, and signal transduction approaches continually enhance the evolution and efficacy of enzyme‐based electrochemical biosensors, ensuring heightened accuracy, increased precision, and applicability in real‐world scenarios [13, 14, 15, 16].

In recent decades, research on conductive polymers has experienced significant growth, largely due to their distinctive molecular structure, which features alternating single and double bonds throughout the molecular backbone. This arrangement enables these materials to conduct electricity, revealing a range of fascinating, intriguing properties, positioning these materials as highly attractive for sophisticated electronic uses. Various electron‐deficient core groups, including derivatives of diketopyrrolopyrroles, quinoxalines, and perylenes, incorporate benzoxazoles and thienopyrroledione (TP) units to reduce LUMO energy levels. This configuration offers sites for substituents, enabling further optimization of electronic, optical, and structural characteristics [17]. Conversely, among the numerous electron‐rich core groups, such as carbazoles, thiophenes, and fluorenes, benzodithiophenes (BDT) are notable for their exceptional donor potential, thanks to their fused ring architecture, effective hole mobility, and available functional positions for enhancing polymer solubility and adjusting properties. Conductive polymers are a remarkable class of materials that fill the gap between conventional insulating polymers and metal conductors [18]. These materials possess distinctive electronic and electrochemical properties, rendering them highly versatile and appealing for diverse applications, such as in sensors, electronics, actuators, and biomedical devices [19]. Additionally, the core of the conducting polymer conjugation concept, which is based on a continuous network of alternating single and double bonds along the polymer backbone, provides additional benefits [20, 21]. Their capacity to conduct electricity, undergo reversible redox reactions, and respond to external factors makes them particularly versatile for applications in these fields [22]. The fundamental conducting polymer feature is the concept of conjugation, which involves a continuous sequence of alternating patterns of single and double bonds across the polymer chain [23, 24]. This conjugation enables the delocalization of π‐electrons, promoting charge transport and electrical conductivity. Unlike standard polymers, conductive polymers can be oxidized or reduced to transition between different electronic states, enhancing their functionality in electronic applications [25, 26].

Quantum dot‐based biosensors represent a revolutionary advancement in detection technologies, harnessing the unique luminescent and conductive features of quantum dots to facilitate superior selectivity and sensitivity in biological molecule identification [27, 28]. Quantum dots are nanoscale semiconductor particles known for their remarkable fluorescence and adjustable electronic features. In biosensing applications, they act as labels or probes, offering significant benefits such as strong, stable fluorescence, broad excitation ranges, and resistance to photobleaching. The interaction between quantum dots and biomolecules is utilized for detection purposes. Quantum dot‐based biosensors find application across diverse sectors such as clinical analysis, ecological monitoring, and bedside diagnostic evaluation [29, 30]. Their key advantages include high sensitivity, multiplexing capabilities (simultaneous detection of multiple targets), and the capacity for real‐time monitoring. Research efforts continue to focus on enhancing quantum dot durability, biocompatibility, and integration of quantum dots in biosensor systems, which are expected to yield increasingly sophisticated and versatile diagnostic technologies. The originality of this study lies in the scarcity of research in the literature on the immobilization of tyrosinase enzymes using functionalized graphene quantum dots, which underscores the significance of this work [31, 32, 33].

Tyrosinase (Tyr) is a pivotal enzyme containing Cu that is involved in melanin production and plays an essential role in various biological processes, particularly in skin and hair pigmentation. This enzyme catalyzes the conversion of tyrosine, a nonpigmented amino acid, into melanin, the coloring agent that determines pigmentation in skin, hair, and ocular tissue. While essential for normal physiological functions, abnormal tyrosinase activity is associated with skin disorders and hyperpigmentation [34, 35]. Regarding tyrosinase, its inhibition has garnered significant attention in both the cosmetic and pharmaceutical industries. Tyrosinase inhibitory compounds are being evaluated for application in dermal lightening products and hyperpigmentation condition management, such as melasma. Numerous naturally occurring and chemically synthesized molecules have been classified as tyrosinase inhibitors, which work by binding to the enzyme's active site or disrupting its cofactors, thereby regulating melanin production [36, 37]. Moreover, catechol serves as the principal Tyr substrate, with the enzyme catalyzing two sequential transformations: monophenol conversion to *o*‐diphenols via monophenolase function, subsequently followed by *o*‐diphenol transformation to *o*‐quinones through diphenolase function, as illustrated in reactions 1 and 2 [38, 39].

Tyrosinase:

(1) $$\mathrm{Catechol}+½O_{2}\to o\text{-}\mathrm{quinone}+H_{2}O$$

(2) $$o\text{-}\text{quinone}+2H^{+}+2e^{-}\to \text{Catechol}$$

Rosmarinic acid, a bioactive compound naturally existing in several plant sources, has generated considerable attention regarding its tyrosinase inhibitory properties. This hydroxylated compound is prevalent in many herbaceous species and is chiefly recognized for its anti‐inflammatory and antioxidative capabilities. Rosemary (*Rosmarinus officinalis L.*), an aromatic, perennial, pale blue, shrub‐like plant from the Lamiaceae family, is cultivated in diverse climates worldwide. In Turkiye, this plant is identified under several common names such as “biberiye,” “akpüren,” “hasalban,” and “kuş dili.” It naturally grows along Turkiye's western and southern coasts, particularly in the regions of Mersin, Tarsus, Adana, Hatay, and Çanakkale [40, 41].

The mechanism by which rosmarinic acid inhibits tyrosinase stems from its interaction with the enzyme's active site, effectively blocking the conversion of tyrosine into melanin. This interference disrupts the melanin synthesis pathway, leading to a reduction in pigment production [42, 43]. The inhibitory action of rosmarinic acid suggests its potential application in formulations designed to lighten the skin, treat hyperpigmentation disorders, and promote an even skin tone, making it a valuable asset for future skincare innovations [44, 45, 46]. Beyond its tyrosinase inhibitory properties, rosmarinic acid also possesses antioxidant and anti‐inflammatory effects, further enhancing its appeal for skincare applications. As research into rosmarinic acid and its role in tyrosinase inhibition continues, this compound holds promise as a natural alternative in the development of cosmetic and dermatological products aimed at addressing skin pigmentation concerns. Its multifaceted benefits make it a compelling candidate for future innovations in the skincare industry [47, 48].

To leverage these advantageous structural configurations, a specifically designed selenium‐bearing random conjugated copolymer, [α‐2‐thienyl‐ω‐2‐thienyl‐poly[4,8‐bis((2‐ethylhexyl)oxy)benzo[1,2‐*b*:4,5‐b′]dithiophene‐*alt*‐(5,6‐dimethoxybenzo[*c* [1, 2, 5] selenadiazole; 5‐(2‐ethylhexyl)‐4*H*‐thieno[3,4‐*c*]pyrrole‐4,6(5*H*)‐dione)] (poly[BDT‐*alt*‐(TP;BSe)]), hereafter referred to as poly[BDT‐alt‐(TP;BSe)], was synthesized and employed as the core electrochemical interface in this study. The rationale for utilizing this specific polymer architecture is multifold. First, the construction of a Donor‐Acceptor (D‐A) backbone, combining the strong electron‐donating BDT units with electron‐deficient TP and benzoselenadiazole (BSe) moieties, effectively narrows the bandgap and lowers the LUMO energy levels. This structural tuning facilitates an ultrafast electron transfer rate between the redox‐active center of the immobilized enzyme and the electrode surface. Second, the incorporation of the heavy chalcogen atom, selenium, into the conjugated backbone significantly enhances the intermolecular interactions due to its larger atomic radius and higher polarizability compared to sulfur. This selenium substitution promotes tighter π‐π stacking and higher intrinsic charge carrier mobility, resulting in an exceptionally conductive matrix. When this highly conductive polymer is coupled with NH_2_‐functionalized graphene quantum dots, it creates a synergistic, large‐surface‐area microenvironment. This specialized interface not only prevents the denaturation of tyrosinase but also provides abundant anchoring sites for stable glutaraldehyde cross‐linking, ultimately yielding a highly sensitive and stable biosensing platform for catechol detection and rosmarinic acid inhibition assays.

Despite the critical need for sensitive inhibitor screening platforms, there is a distinct scarcity of research exploring the synergistic use of functionalized graphene quantum dots and novel heteroatom‐doped conducting polymers for tyrosinase immobilization. To bridge this gap, the primary aim of this study is to design, fabricate, and systematically evaluate a novel multifunctional amperometric nanobiosensor (GE/poly[BDT‐alt‐(TP;BSe)]/NH_2_ *f*QDots‐Tyr) tailored for the highly sensitive quantification of catechol and the subsequent screening of tyrosinase inhibition by rosmarinic acid. The fabricated biosensor is electrochemically characterized through cyclic voltammetry (CV) and chronoamperometry (CA). Every condition affecting the biosensor's response, including the amounts of tyrosinase, poly[BDT‐alt‐(TP;BSe)], NH_2_ *f*QDots, and glutaraldehyde, as well as inhibition time and concentration, was optimized. The inhibition study was assessed using the chronoamperometric method by quantifying the decrease in the catechol signal upon exposure to rosmarinic acid, which was obtained from *Rosmarinus officinalis* plant.

## 2 Results and Discussion

### 2.1 Optimization Studies of GE/Poly[BDT‐alt‐(TP;BSe)]/NH_2_ *f*QDots‐Tyr Nanobiosensor

**FIGURE 1:**
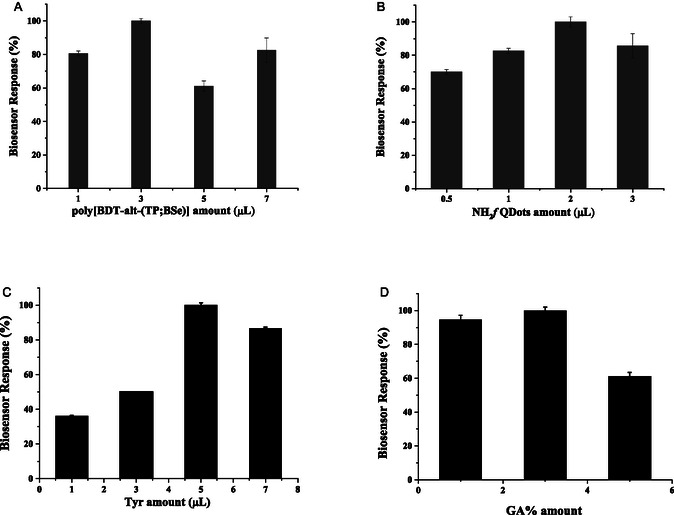
The optimization process involved adjusting the quantities of (A) poly[BDT‐alt‐ (TP;BSe)], (B) NH_2_ *f*QDots, (C) Tyrosinase, and (D) Glutaraldehyde. These optimizations were carried out under agitation at 250 rpm, with a working potential of −0.2 V in 0.1 M phosphate buffer containing 0.1 M KCl at pH 6.5.

Before commencing the detection and inhibition analyses, the biosensor interface was optimized using a sequential, one‐factor‐at‐a‐time (OFAT) approach. In this systematic method, a single parameter was varied while all others were kept constant at their initially estimated or previously optimized values. The optimization sequence was rationally designed from the bottom up, prioritizing the foundational electroactive layer before moving to the biological recognition elements. Several parameters, including NH_2_fQDs, poly[BDT‐alt‐(TP;BSe)], Tyr, and glutaraldehyde amounts, were optimized. Among all the investigated parameters, the amount of the conducting polymer, poly[BDT‐alt‐(TP;BSe)], was identified as the most relevant and critical factor dictating the overall biosensor performance. Because it constitutes the foundational electrocatalytic interface, its precise volume directly governs the surface conductivity, the active surface area available for NH_2_‐GQD anchoring, and the ultimate electron transfer efficiency between the enzyme's redox center and the electrode. As observed, adjusting the poly[BDT‐alt‐(TP;BSe)] volume from 1 to 7 μL showed that 3 μL yielded the highest catechol response (Figure 1A). Volumes below this threshold provided insufficient surface coverage and sluggish electron transfer, while excessive volumes likely led to the formation of a thick, resistive polymer film that hindered mass transfer and electron tunneling. Following the optimization of the foundational layer, the sequential optimization continued with the concentration of NH_2_‐f‐QDs. By testing volumes ranging from 0.5 to 3 μL on the optimized 3 μL poly[BDT‐alt‐(TP;BSe)] layer, 2 μL of NH_2_‐f‐QDs provided optimal signal amplification (Figure 1B). Subsequently, the quantity of the Tyr enzyme was evaluated (spanning from 1 to 7 μL). It was found that 5 μL of Tyr was optimal for sensing CAT (Figure 1C); depositing excessive Tyr resulted in improper nanocomposite matrix formation, as the surplus protein could not efficiently crosslink. Finally, the glutaraldehyde concentration was optimized in the same sequential manner, with 2.5% glutaraldehyde providing the ideal balance between structural integrity and enzyme activity (Figure 1D).

### 2.2 Surface Morphological Characterizations of GE/Poly[BDT‐alt‐(TP;Bse)]/NH_2_ *f*QDots‐Tyr Nanobiosensor

**FIGURE 2:**
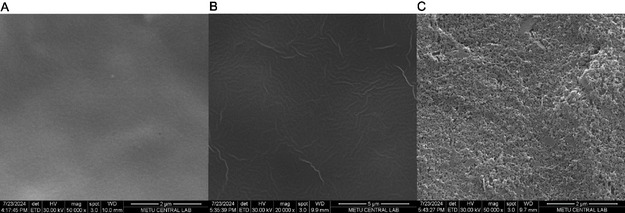
SEM images of (A) Bare GE, (B) poly[BDT‐alt‐(TP;BSe)], and (C) GE/poly[BDT‐alt‐(TP;BSe)]/NH_2_ *f*QDots‐Tyr modified electrodes.

Scanning electron microscopy (SEM) was utilized to examine the morphological changes of the electrode surface throughout the successive modification step, as presented in Figure 2. First, the SEM micrographs of the bare graphite electrode and the electrode coated solely with poly[BDT‐alt‐(TP;BSe) conducting polymer are clearly distinguished in Figure 2A,B, respectively. As shown in Figure 2B, the conducting polymer clusters, of varying sizes, are uniformly and well dispersed across the polymer‐coated electrodes. The surface formed after the immobilization of Tyr is observed in Figure 2C. Following the biomolecule integration, the surface morphology has significantly changed, resulting in a much rougher texture compared to the surface prior to the pre‐enzyme state.

### 2.3 Electrochemical Characterization of the GE/Poly[BDT‐alt‐(TP;BSe)]/NH_2_ *f*QDots‐Tyr Nanobiosensor

**FIGURE 3:**
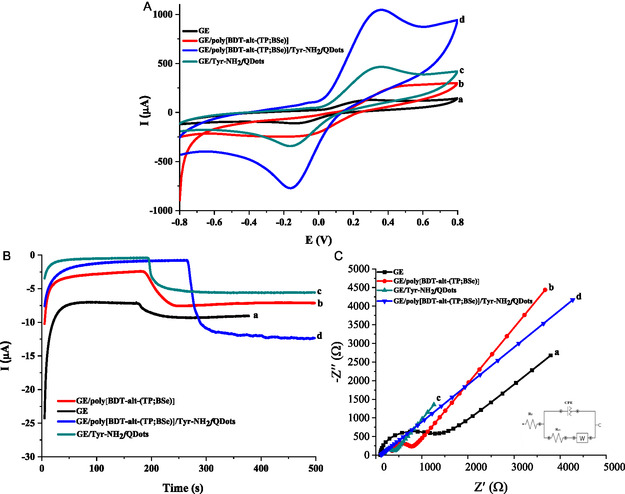
(A) Cyclic voltammograms obtained for (a) GE, (b) GE/poly[BDT‐alt‐(TP;BSe)], (c) GE/Tyr‐NH_2_ *f*QDs, and (d) GE/poly[BDT‐alt‐(TP;BSe)]/NH_2_ *f*QDots‐Tyr in the presence of catechol in 50 mM phosphate buffer at pH 6.5 with 0.1 M KCl. (B) Current‐time responses of (a) GE, (b) GE/poly[BDT‐alt‐(TP;BSe)], (c) GE/Tyr‐NH_2_ *f*QDs, and (d) GE/poly[BDT‐alt‐(TP;BSe)]/NH_2_ *f*QDots‐Tyr upon sequential additions of 5 mM catechol under stirring conditions (250 rpm) at an applied potential of −0.2 V in 50 mM phosphate buffer pH 6.5 supplemented with 0.1 M KCl. (C) Fitted EIS of (a) GE (b) GE/poly[BDT‐alt‐(TP;BSe)], (c) GE/Tyr‐NH_2_ *f*QDs, and (d) GE/poly[BDT‐alt‐(TP;BSe)]/NH_2_ *f*QDots‐Tyr in 5.0 mM K_3_[Fe(CN)_6_]/K_4_[Fe(CN)_6_] containing 0.1 M KCl in 50 mM phosphate buffer at pH 7.0.

The electrochemical properties of GE/poly[BDT‐alt‐(TP;BSe)]/NH_2_ *f*QDots‐Tyr nanobiosensors were initially characterized by using CV. Figure 3A illustrates that the overlaid cyclic voltammograms depict the step‐by‐step modification of the designed nanobiosensor for detecting 50 µM CAT. Additionally, chronoamperometric analyses were performed using 1.25 mM catechol with various electrode configurations GE, GE/poly[BDT‐alt‐(TP;BSe)], GE/Tyr‐NH_2_ *f*QDots, GE/poly[BDT‐alt‐(TP;BSe)]/Tyr‐NH_2_ *f*QDots, and to assess the impact of sensor modifications on the substrate's response. Moreover, the amperometric results presented in Figure 3B prove that the catechol response was enhanced following the modification with GE/poly[BDT‐alt‐(TP;BSe)]/NH_2_ *f*QDots‐Tyr. Distinct and well‐defined reduction peaks were detected at −200 mV for the modified electrodes in 50 mM PBS pH 6.5, in accordance with previously reported catecholase activity [49]. The inclusion of an extra conductive GQDs layer onto the electrode surface resulted in a relatively higher current response for CAT using GE/poly[BDT‐alt‐(TP;BSe)]/NH_2_ *f*QDots‐Tyr as shown in the blue‐colored line, compared to a solely NH_2_ *f*QDs‐modified electrode. Furthermore, the highly conductive nature of the designed polymer significantly accelerated the electron transfer rate, leading to an enhanced current response for CAT compared to the GE/Tyr‐NH_2_ *f*QDs configuration. Moreover, electrochemical impedance spectroscopy (EIS) is a highly insightful technique to monitor the step‐by‐step interfacial changes during electrode functionalization. EIS studies were performed in 5.0 mM K_3_[Fe(CN)_6_]/K_4_[Fe(CN)_6]_ containing 0.1 M KCl in 50 mM phosphate buffer at pH 7.0. The Nyquist plots clearly demonstrate the sequential changes in charge transfer resistance at each modification step and are added as Figure 3C.

### 2.4 Analytical Characterization of GE/Poly[BDT‐alt‐(TP;BSe)]/NH_2_ *f*QDots‐Tyr Nanobiosensor

**FIGURE 4:**
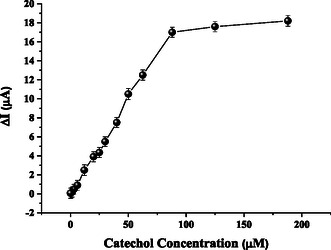
The analytical calibration of the GE/poly[BDT‐alt‐(TP;BSe)]/NH_2_ *f*QDots‐Tyr biosensor was conducted by plotting the current response against various catechol concentrations. The inset presents representative current‐time profiles obtained during successive catechol additions to the electrochemical cell. These measurements were taken under stirring conditions at an applied potential of −200 mV in a 0.1 M phosphate buffer containing 0.1 M KCl at pH 6.5.

Following parameters optimization, chronoamperometric evaluation of catechol detection was performed using the fabricated nanobiosensor, the GE/poly[BDT‐alt‐(TP;BSe)]/NH_2_ *f*QDots‐Tyr. A linear response toward catechol was obtained over the concentration range of 0.1–88 µM, as shown in the Figure 4, consistent saturated response for catechol was achieved at concentrations above 90 µM, represented by the equation *y* = 0.1975*x*−0.1109(*R* ^2^ = 0.996). Amperometric responses to varying catechol concentrations obtained with the nanobiosensor are illustrated in Figure 4.

Furthermore, key analytical parameters were calculated, including the limit of detection (LOD) and limit of quantification (LOQ), estimated using the expressions 3σ/S and 10σ/S, respectively, where σ represents the standard deviation of the blank response, and S denotes the slope of the calibration plot. The LOD was found to be 0.023 µM, while the LOQ was obtained to be 0.007 µM.

The analytical performance of the developed GE/poly[BDT‐alt‐(TP;BSe)]/NH_2_ *f*QDots‐Tyr nanobiosensor for catechol detection was compared with existing electrochemical transducers previously reported in published studies, as shown in Table 1. The proposed nanobiosensor demonstrates a broader linear detection range alongside a competitive LOD value when compared to existing literature. It offers a superior linear dynamic range compared to its peers, making it highly effective for applications requiring the quantification of higher concentrations of phenolic compounds without extensive sample dilution.

**TABLE 1:** Comparison of some selected conducting polymer and quantum dot modified tyrosinase based sensors for detection of various phenolic compounds.

Sensor	Phenolic compound	Medium	Method	Linear range	LOD	App.	References
Tyr/Fe^2+^‐PPY/ITO	Phenol	0.2 M PBS (pH 7.0)	CA	9.9–84.7 μM	2.90 μM	NS^a^	
	Catechol			6.7–72.6 μM	2.03 μM		[50]
	*p*‐Chlorophenol			3.9–48.8 μM	1.19 μM		
Tyr/Pt/PANI	Catechol	B–R (pH 6.0)	CA	0.2–80 μM	NS^a^	NS^a^	[51]
Tyr–ZnOQDs/GO/GCE	Hydroxylated polychloro‐biphenyls	0.1 M PBS (pH 7.2)	SWV	2.8–27.65 μM	0.15 μM	Wastewater samples	[52]
CdSQDs/Chit/Tyr	Catechol	0.1 M PBS (pH 6.5)	CA	1.0 nM–20 µM	0.3 nM	Jinshan lake, tap water	[53]
	*p‐*Cresol			1.0 nM–20 µM	0.5 nM		
	Phenol			6.5 nM–30 µM	1.0 nM		
	*m‐*Cresol			10 nM–15 µM	2.5 nM		
	*p*‐Chlorophenol			15 nM–8.0 µM	4.0 nM		
CTAB‐NCC/QDs/Tyr/SPCE	Phenol	0.1 M PBS (pH 7.0)	DPV	5–40 μM	0.082 μM	Lake water	[54]
	Catechol			1–25 μM	0.125 μM		
	*o*‐Cresol			2–10 μM	0.007 μM		
	4‐Chlorophenol			5–25 M	0.021 μM		
SPCE/GQDs@PEDOT NPs/Tyr	Catechol	0.1 M PBS (pH 6.5)	CA	0.005–11 μM	0.002 μM	Pharmaceutical dosage forms	[43]
	Epinephrine			0.2–12 μM	0.065 μM		
	Norepinephrine			0.1–2.5 μM	0.035 μM		
GE/poly[BDT‐alt‐(TP;BSe)]/NH_2_ *f*QDots‐Tyr	Catechol	1. M PBS (pH 6.5)	CA	1–88 µM	0.023 μM	Inhibition	**This study**

a Not Stated;B–R: Britton–Robinson; CA: Chronoamperometry; Chit : Chitosan; CTAB: Cetyltriammonium Bromide; DPV: Differential Pulse Voltammetry; G: Graphene; GCE: Glassy Carbon Electrode; GE: Graphite electrode; GO: Graphene Oxide; ITO: Indium Tin Oxide; NCC: Nanocrystalline cellulose; NPs: Nanoparticles; PANI: Polyaniline; PBS: Phospahate Buffer Solution; PEDOT: Poly (3,4‐ethylenedioxythiophene); PPY: Hexacyanoferrate(II)‐ion‐doped Conducting Polypyrrole; QDs: Quantum Dots; SPCE: Screen Printed Carbon Electrode; SWV: Square Wave Voltammetry; Tyr: Tyrosinase; ZnO: Zinc Oxide.

### 2.5 Repeatability, Reproducibility, and Stability of the GE/Poly[BDT‐alt‐(TP;BSe)]/NH_2_ *f*QDots‐Tyr Nanobiosensor and Interference Studies

To evaluate repeatability, five successive amperometric measurements were performed using a single GE/poly[BDT‐alt‐(TP;BSe)]/NH_2_ *f*QDots‐Tyr nanobiosensor for the detection of 50 µM catechol in a 50 mM phosphate buffer at pH 6.5. Additionally, the reproducibility of this sensor was evaluated by the electrochemical responses of freshly constructed nanobiosensors across multiple preparation batches, using the same concentration of catechol in the same buffer. The reported low relative standard deviation (RSD%) values indicate that the developed nanobiosensor exhibits both repeatability and reproducibility.

**FIGURE 5:**
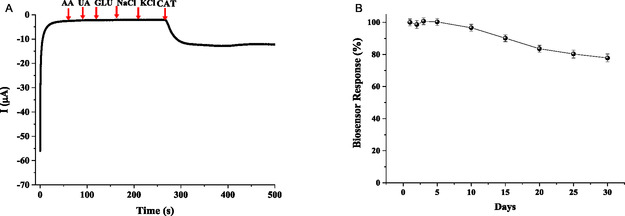
(A) Interference effects observed in the GE/poly[BDT‐alt‐(TP;BSe)]/NH_2_ *f*QDots‐Tyr nanobiosensor. (B) Stability of the GE/poly[BDT‐alt‐(TP;BSe)]/NH_2_ *f*QDots‐Tyr nanobiosensor in 30 days. Experimental conditions: 50 µM catechol concentration at an applied potential of −200 mV in a 50 mM phosphate buffer solution at pH 6.5.

The selectivity of the designed nanobiosensor was confirmed by examining the influence of the interference from endogenous substances commonly occurring in physiological matrices, including glucose, ascorbic acid, uric acid, NaCl, and KCl, on the analytical signal. These compounds were introduced into the buffer at 1 mM concentrations in a 1:1 ratio with the substrate prior to the addition of CAT. The absence of any significant alteration in the signal indicated that the nanobiosensor maintained high selectivity toward Cat detection, even though these ions were present (Figure 5A).

Furthermore, the nanobiosensor was examined in terms of operational stability and shelf‐life behavior. Nanobiosensors were stored at 4°C and monitored daily over 30 consecutive days for shelf‐life evaluation. After 10 days, the biosensors retained 96.71% of their initial activity, and after 30 days, they maintained 77.86% of their activity (Figure 5B). These findings suggest that the nanobiosensor could be used as a disposable tool. Therefore, the nanobiosensor demonstrates consistent long‐term stability for daily usage in detecting CAT.

### 2.6 Inhibition Studies of Rosmarinic Acid on GE/Poly[BDT‐alt‐(TP;BSe)]/NH_2_ *f*QDots‐Tyr Nanobiosensor

**FIGURE 6:**
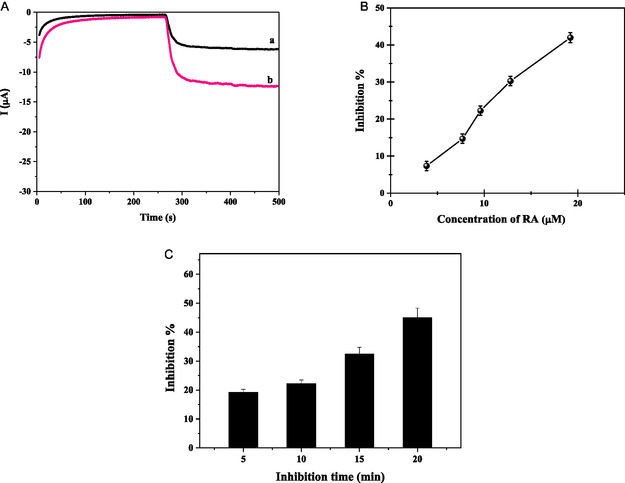
Continue‐(A) Current‐time responses of the GE/poly[BDT‐alt‐(TP;BSe)]/NH_2_ *f*QDots‐Tyr nanobiosensor, (a) after inhibition and (b) before inhibition. (B) Calibration plot depicting the relationship between inhibition percentage and rosmarinic acid concentration. (C) Time‐dependent inhibition profile for the developed nanobiosensor. Experimental conditions: Working potential of −200 mV in 50 mM phosphate buffer at pH 6.5.

The inhibition studies were carried out using the ethanolic extract of *Rosmarinus officinalis*, which contains rosmarinic acid. The rosmarinic acid concentration was measured by HPLC and was 1.92 mM. The GE/poly[BDT‐alt‐(TP;BSe)]/NH_2_ *f*QDots‐Tyr nanobiosensor was employed to electrochemically evaluate the inhibitory action of rosmarinic acid on Tyr activity. The nanobiosensor's response to catechol, as shown in Figure 6, was observed both before and after the inhibition of tyrosinase. Initially, the inhibition time of rosmarinic acid was investigated by testing between 5 and 20 min, and it was concluded that tyrosinase exhibited consistent inhibition within 10 min, which was then used for further steps. Subsequently, rosmarinic acid's concentration‐dependent inhibitory behavior was examined across 4–19 µM. Utilizing the developed nanobiosensor, the inhibition effect of rosmarinic acid could be monitored with a LOD of 0.134 µM and a LOQ of 0.41 µM. Moreover, the inhibitory strength was evaluated by calculating the *I*_50_ value, defined as the inhibitor concentration producing 50% reduction in enzymatic activity at a fixed substrate concentration, with a determined value of 21 µM.

## 3 Conclusion

Comprehending and harnessing tyrosinase inhibition opens up valuable opportunities for developing new therapeutic treatments and cosmetic products aimed at managing skin pigmentation. The commercialization of such inhibitors holds promising prospects in both the medical and beauty industries for controlling pigmentation‐related disorders. Conductive polymer‐based biosensors are at the cutting edge of innovation, combining the distinctive features of conductive polymers with the biological recognition elements' specificity. The interface between the conductive polymer and biological recognition components, which is often an enzyme or antibody, enables the direct conversion of biological events into electrical signals. The outstanding sensitivity and selectivity of conductive polymers make them ideal for biosensor applications. Their ability to undergo reversible redox reactions and modify their electrical properties when interacting with specific biomolecules allows for accurate monitoring of various analytes, from glucose and DNA to proteins and neurotransmitters. This versatility positions conductive polymer‐based biosensors as essential tools in medical diagnostics, environmental assessment, and food safety. Additionally, the incorporation of conductive polymers into biosensors facilitates portable, rapid, and cost‐effective diagnostic device development. Current research focuses on improving the stability, biocompatibility, and specificity of these biosensors, paving the way for groundbreaking advances that could transform healthcare and other sectors.

The substitution of sulfur with selenium, an atom with a larger atomic radius and higher polarizability, in the polymer backbone enhances intermolecular interactions and π‐π stacking. This maximizes charge carrier mobility and overall conductivity, which are critical for the sensitivity of the biosensor. The combination of electron‐donating groups like BDT with electron‐accepting groups such as TP and BSe narrows the HOMO‐LUMO energy gap of the polymer. This facilitates a much faster transfer of electrons generated during the catalytic reaction of the tyrosinase enzyme to the electrode surface. Moreover, when combined with NH_2_‐GQDs, this specific polymer creates a highly conductive and biocompatible 3D network where the tyrosinase can be immobilized without losing its native structure. The cross‐linking performed via glutaraldehyde with the amine groups on the GQDs significantly enhances the mechanical and electrochemical stability of the interface.

In light of this information, in this study, the inhibition effects of rosmarinic acid on tyrosinase enzyme by designing selenium‐bearing conjugated polymer and amine‐functionalized graphene quantum dots based electrochemical nanobiosensor were examined, and the results obtained may be important in the field of health sciences, with the idea that it may carry suggestions that may be pioneering for the development of new drug formulations. Studies have shown that rosmarinic acid exerts inhibitory effects on tyrosinase activity, positioning it as a promising candidate for use in skincare and cosmetic formulations. The originality of this study lies in the scarcity of research in the literature on the immobilization of tyrosinase enzymes using functionalized graphene quantum dots, which underscores the significance of this work. It is thought that the deficiency in the literature on this subject has been filled. These features enrich the originality of this study. Moreover, under optimized conditions, a LOD of 23 nM and LOQ of 7 nM were achieved for catechol detection. These results highlight the sensor's sensitivity. In addition, the fact that there are no electrochemical nanobiosensor studies on the inhibition effects of Rosmarinic Acid in the literature is important for the originality of the study. Rosmarinic acid has emerged as a promising compound for cosmetic and pharmaceutical formulation use due to its skin‐whitening potential, which is attributed to its tyrosinase inhibitory activity. Here, tyrosinase inhibition by rosmarinic acid was evaluated using extracts from selected plant species known to contain this bioactive compound. The developed electrochemical nanobiosensor platform demonstrates significant potential for broader applications, including the immobilization of various enzymes and the assessment of enzyme inhibition by different pharmaceutical agents. Beyond its tyrosinase inhibitory properties, rosmarinic acid also possesses antioxidant and anti‐inflammatory effects, further enhancing its appeal for skincare applications. As research into rosmarinic acid and its role in tyrosinase inhibition continues, this compound holds promise as a natural alternative in the development of cosmetic and dermatological products.

## 4 Experimental

### 4.1 Apparatus

In each CA and CV measurement, measurements were conducted using a PalmSens4 Potentiostat / Galvanostat / Impedance Analyzer, presented with PSTrace Version 5.8 software package. A three‐electrode configuration employed a graphite rod electrode (counter‐flat top, 3.05 mm dia, 38.10 mm long, 99.9995% metal basis 122, Alfa Aesar GmbH&Co KG, Karlsruhe, Germany) as the working electrode, Ag/AgCl (BASi; 3 M NaCl) as the reference electrode, and platinum wire as the auxiliary electrode. Amperometric measurements were performed in triplicate, and averages were reported. The Agilent Hewlett‐Packard1100 HPLC system (Avondale, USA) consisted of a DAD variable wavelength detector and data processing, supported by Chemstation B.04.03 version, which was employed for rosmarinic acid levels of *Rosmarinus officinalis* plant. All measurements were run at room temperature. All electrochemical experiments were conducted at room temperature. In addition, chronoamperometric measurements were conducted under stirring conditions at a constant potential of −200 mV. SEM images were captured on each modified surface with different combinations using JEOL JSM‐6400 instruments.

### 4.2 Reagents

Mushroom Tyrosinase (≥1000 unit/mg), catechol, glutaraldehyde (GA, 25%), potassium phosphate monobasic (KH_2_PO_4_), potassium phosphate dibasic (K_2_HPO_4_), potassium chloride (KCl), ascorbic acid, uric acid, glucose, and sodium chloride were obtained from Sigma–Aldrich (St. Louis, MO, USA). Aqueous NH_2_ functionalized graphene quantum dots (DRP‐GQD: λem = 495 nm; concentration = 15 mg/mL; diameter Ø = 100 nm) were acquired from DropSens (Llanera, Spain). Solutions were formulated using ultrapure water from a Millipore‐Milli‐Q system (Merck KGaA, Darmstadt, Germany) and maintained at 4°C when not in use.

### 4.3 Synthesis of Conducting Polymer (poly[BDT‐alt‐(TP;BSe)])

Three moieties of BSe, TP, and benzodithiophene containing selenium‐bearing random conjugated polymer named [α‐2‐thienyl‐ω‐2‐thienyl‐poly[4,8‐bis((2‐ethylhexyl)oxy)benzo[1,2‐*b*:4,5‐b′]dithiophene‐*alt*‐(5,6‐dimethoxybenzo[*c* [1, 2, 5] selenadiazole; 5‐(2‐ethylhexyl)‐4*H*‐thieno[3,4‐*c*]pyrrole‐4,6(5*H*)‐dione)] (poly[BDT‐*alt*‐(TP;BSe)]) was synthesized following a previously established protocol [55] and directly used. In brief, a solution was prepared by dissolving 1,3‐Dibromo‐5‐(2‐ethylhexyl)‐4*H*‐thieno[3,4‐*c*]pyrrole‐4,6‐(5*H*)‐dione (70 mg, 0.197 mmoL, 1 eq), 4,7‐dibromo‐5,6‐dimethoxybenzo[*c* [1, 2, 5] thiadiazole (84 mg, 0.197 mmoL, 1 eq), and 2,6‐bis(trimethylstannyl)−4,8‐bis(2‐ethylhexyloxy)benzo[1,2‐*b*:4,5‐*b’*]dithiophene (305 mg, 0.396 mmol, 2 eq) in toluene (15 mL) under an inert atmosphere. Following 20 min of nitrogen gas bubbling, Pd(PPh_3_)_2_Cl_2_ (5% moL) was added, and the reaction underwent reflux for 48 h. Subsequently, end cappers stannylated thiophene and bromo thiophene were introduced to the reaction in 4 eq and 8 eq amounts, respectively, for 6 h and overnight. The polymer was then precipitated in methanol (100 mL) the next day. The resulting crude solid product underwent purification through Soxhlet extraction using methanol, acetone, hexane, and chloroform sequentially. The chloroform fraction was isolated and treated with quadrasil 10–20 mg, followed by stirring for 3 h. The solution was subsequently filtered through a silica plug, and solvent evaporation under vacuum afforded a viscous polymer solution. Precipitation in methanol (100 ml and filtration provided the solid polymer product with an 84% yield. Gel permeation chromatography (GPC) revealed Mn = 40.6 kDa, Mw = 579.9 kDa, and PDI = 14.3. Thermogravimetric analysis (TGA) indicated a decomposition temperature of 95% at 335°C. Differential scanning calorimetry (DSC) showed no distinctive thermal behavior in the range of 26°C to 250°C as reported [55].

### 4.4 Preparation of Solutions

100 mM phosphate buffer solution (PBS) supplemented with KCl was formulated using monobasic and dibasic potassium phosphates. The conducting polymer [*α*‐2‐thienyl‐*ω*‐2‐thienyl‐poly[4,8‐bis((2‐ethylhexyl)oxy)benzo[1,2‐*b*:4,5‐*b*′]dithiophene‐*alt*‐(5,6‐dimethoxybenzo[*c* [1, 2, 5] selenadiazole;5‐(2‐ethylhexyl)‐4*H*‐thieno[3,4‐*c*]pyrrole‐4,6(5*H*)‐dione)] (poly[BDT‐*alt*‐(TP;BSe)]) was prepared trough dispersing 1 mg of the polymer in 1 mL CHCl_3_. For the enzyme preparation, Tyr solution (1 mg/50 μL, 8503 Units per mg solid) was formulated by dissolving the enzyme in 50 mM KCl‐free PBS (pH 6.50). An aliquot of 5 μL (850 units) of the Tyr solution was immobilized onto GE/poly[BDT‐alt‐(TP;BSe)] and subsequently crosslinked using 5 µL of 2.5% glutaraldehyde solution. The GA solution was obtained by diluting a 25% GA with ultrapure water. Catechol was dissolved in PBS to obtain a 25 mM stock solution of catechol. Rosmarinic acid samples were extracted according to the following procedure: The dried aerial plant material was ground into a fine powder (20g) and subjected to maceration in 70% EtOH (200 ml) at room temperature for 24 h, followed by an ultrasonication for 1 h, The mixture was subsequently filtered. This extraction cycle was repeated five times, adding fresh solvent to the residue each time. The filtrates were pooled and concentrated to dryness in vacuo, and the resulting solid extract was kept at 4°C until needed.

### 4.5 Preparation of GE/Poly[BDT‐alt‐(TP;BSe)]/NH_2_ *f*QDots‐Tyr Nanobiosensor

Preceding the utilization, graphite rod electrodes were mechanically polished using emery paper, followed by thorough rinsing with ultrapure water, and allowed to air dry. Subsequently, the nanobiosensor was developed by depositing 3 μL of poly[BDT‐alt‐(TP;BSe) solution, then it was kept on hold and washed with ultrapure water. Later on, a 2 μL of NH_2_ functionalized quantum dots solution was added onto poly[BDT‐alt‐(TP;BSe) GE/poly[BDT‐alt‐(TP;BSe)] nanobiosensor together with 5 μL Tyr. Then, for crosslinking, 5 µL of 2.5% glutaraldehyde solution was utilized. Fabricated GE/poly[BDT‐alt‐(TP;BSe)/NH_2_ *f*QDots‐Tyr nanobiosensor was allowed to dry at room temperature for 2 h before taking amperometric measurements of catechol biosensing.

### 4.6 Monitoring the Tyr Inhibition via Detection of Catechol

Catechol biosensing was evaluated using CA at room temperature with continuous stirring, maintaining a fixed potential of 200 mV. For this purpose, an electrochemical cell was set up, incorporating the GE/poly[BDT‐alt‐(TP;BSe)]/NH_2_ *f*QDots‐Tyr nanobiosensor as the working electrode, an Ag/AgCl reference electrode (BASi; 3 M NaCl), and a platinum wire as the auxiliary electrode. All the measurements were performed in 50 mM phosphate buffer (pH 6.5, 10 mL), with a fresh buffer solution used for each measurement cycle. Once a steady state was achieved, catechol was introduced into the electrolyte solution, allowing for the monitoring of the response to 50 μM catechol.

After preparation and optimization of GE/poly[BDT‐alt‐(TP;BSe)]/NH_2_ *f*QDots‐Tyr nanobiosensor, Tyr inhibition by Rosmarinic Acid was examined. In order to assess the inhibition dynamics as a function of time and concentration of Tyr, the ethanolic extract of Rosmarinus officinalis, which contains rosmarinic acid, was used and progressively diluted in phosphate buffer to establish a range of working concentrations. Initially, baseline catechol measurements were performed on all freshly prepared electrodes to establish their initial activity. These baseline signals were recorded as “I_o_” for each electrode individually. After careful rinsing with distilled water, the electrodes were subsequently immersed in tubes containing 10 µM Rosmarinic Acid, varying inhibition times ranging from 5 to 20 min to assess the inhibition of Tyr by Rosmarinic Acid. After the procedure of the inhibition of the electrode, another amperometric measurement was conducted again, which was referred to as “I” value. Long story short, CA measurements for catechol were conducted again for each immersed and inhibited nanobiosensor, the inhibitory impact of Rosmarinic Acid on Tyr was assessed by monitoring the decrease in catechol signal. The degree of Tyr enzyme inhibition by Rosmarinic Acid was quantified by determining the percentage reduction in catechol current response represented by the equation below,

$$\%\;I=\frac{I_{0}‐I_{1}}{I_{0}}\times 100$$

## Funding

This study was supported by Ankara Universitesi (BAP‐TYL‐2024‐3770).

## Conflicts of Interest

The authors declare no conflicts of interest.

## Data Availability

The data that support the findings of this study are available from the corresponding author upon reasonable request.
